# Supplementary material for: Substrate engagement by the intramembrane metalloprotease SpoIVFB
Source: Nat Commun. 2024 Oct 17;15:8276. doi: 10.1038/s41467-024-52634-6 (PMC11486902; doi:10.1038/s41467-024-52634-6)
Supplement: Supplementary file 1 — Supplementary Information [file 41467_2024_52634_MOESM1_ESM.pdf]

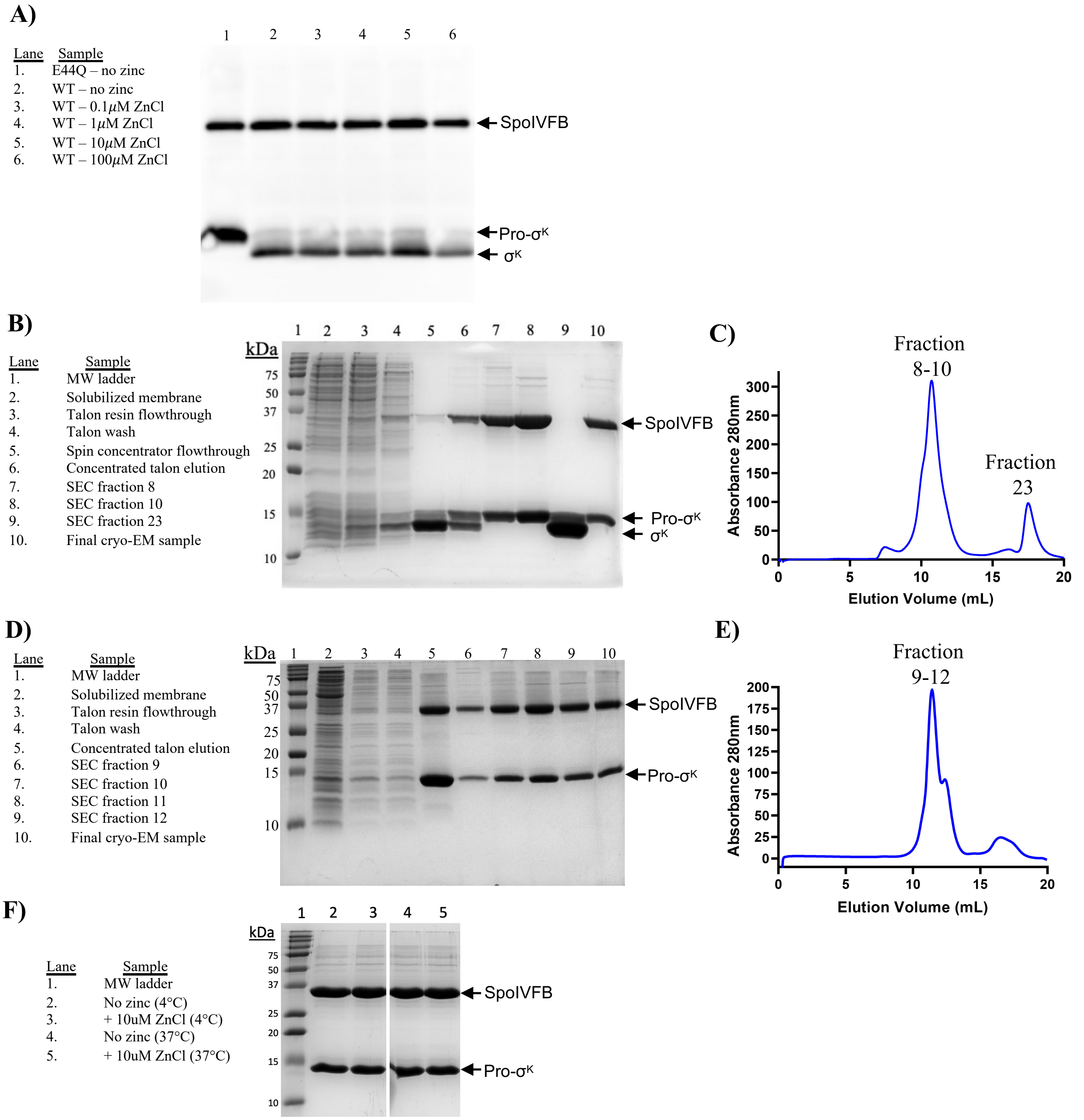

Supplemental Figure S1. WT and E44Q SpoIVFB Co-purify with Pro-σ^K in Large Protein Complexes

A) Anti-pentaHis western blot showing cleavage of Pro-σ^K when exogenous zinc is supplemented to the culture media at time of induction. Zinc supplementation has no effect on the ability of SpoIVFB to cleave Pro-σ^K when the proteins are co-expressed in *E. coli*. Addition of ZnCl at concentrations of 1mM and above was detrimental to bacterial growth. **B)** SDS-PAGE analysis of fractions obtained throughout purification of the WT SpoIVFB and Pro-σ^K preparation. Samples loaded into each lane are indicated in the table to the left. **C)** Gel-filtration chromatogram of detergent solubilized WT SpoIVFB and Pro-σ^K. Fractions from each peak that are loaded on the gel in **B** are indicated. **D)** SDS-PAGE analysis of fractions obtained throughout purification of the E44Q SpoIVFB and Pro-σ^K. Samples loaded into each lane are indicated in the table to the left. **E)** Gel-filtration chromatogram of detergent solubilized E44Q SpoIVFB and Pro-σ^K. Fractions from each peak that are loaded on the gel in **D** are indicated. **F)** WT SpoIVFB lacks *in vitro* activity after detergent solubilization and purification. Purified SpoIVFB:Pro-σ^K samples were incubated for 5 hours with or without ZnCl at 4°C or 37°C. None of the samples show cleavage of Pro-σ^K.

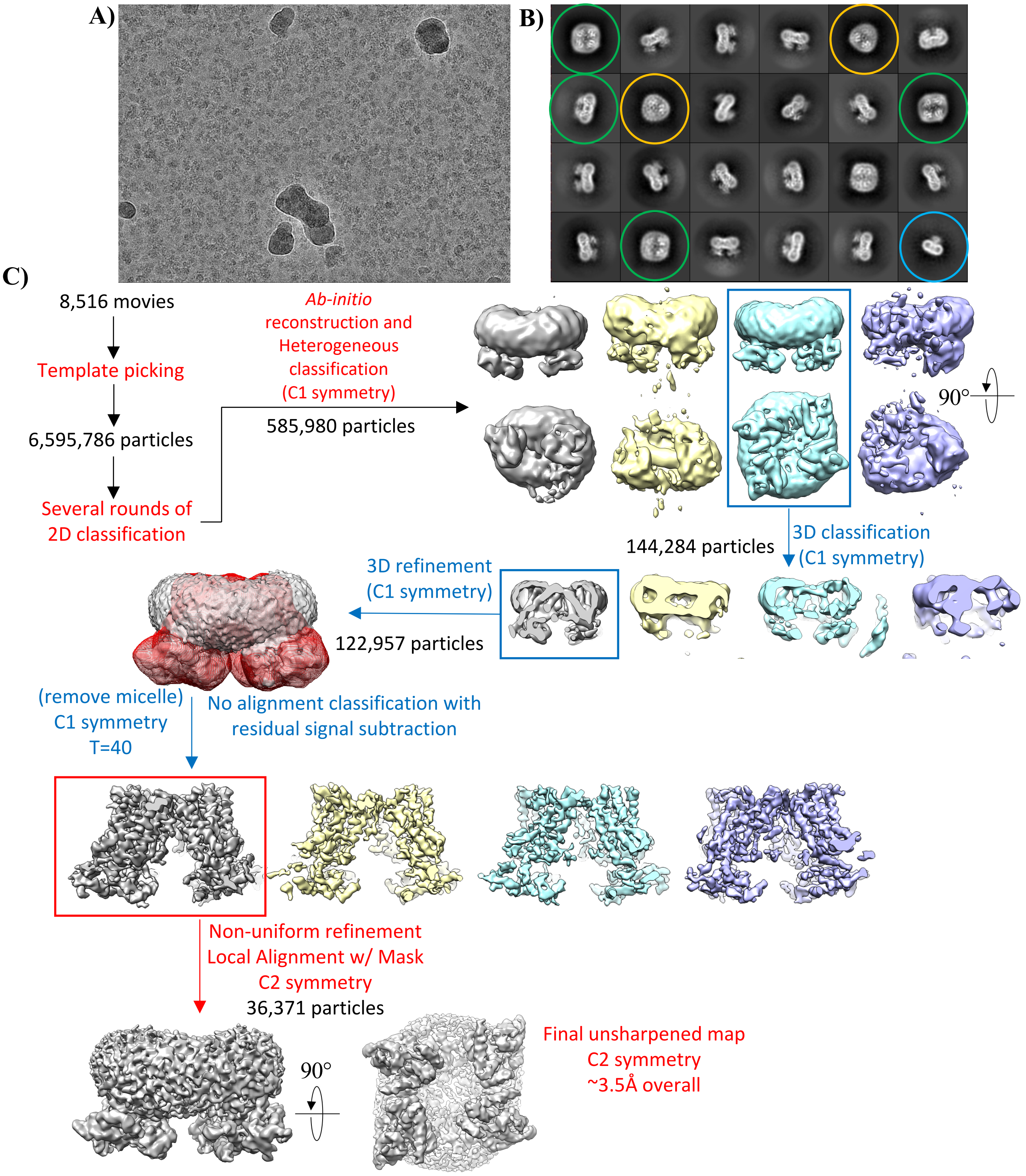

Supplemental Figure S2. Cryo-EM Processing for WT SpoIVFB Co-purified with Pro-σ^K **A)** Representative electron micrograph from a dataset of 8,516 micrographs showing the distribution of particles in ice. **B)** Representative 2D class-averages. Circled averages show examples of SpoIVFB:Pro-σ^K tetramers (green), trimers (orange), and dimers (blue). **C)** Data processing scheme to reconstruct the tetrameric assembly of SpoIVFB:Pro-σ^K complexes. Steps shown in red were performed in CryoSPARC and steps shown in blue were performed in Relion. Particle numbers in each step are written in black.

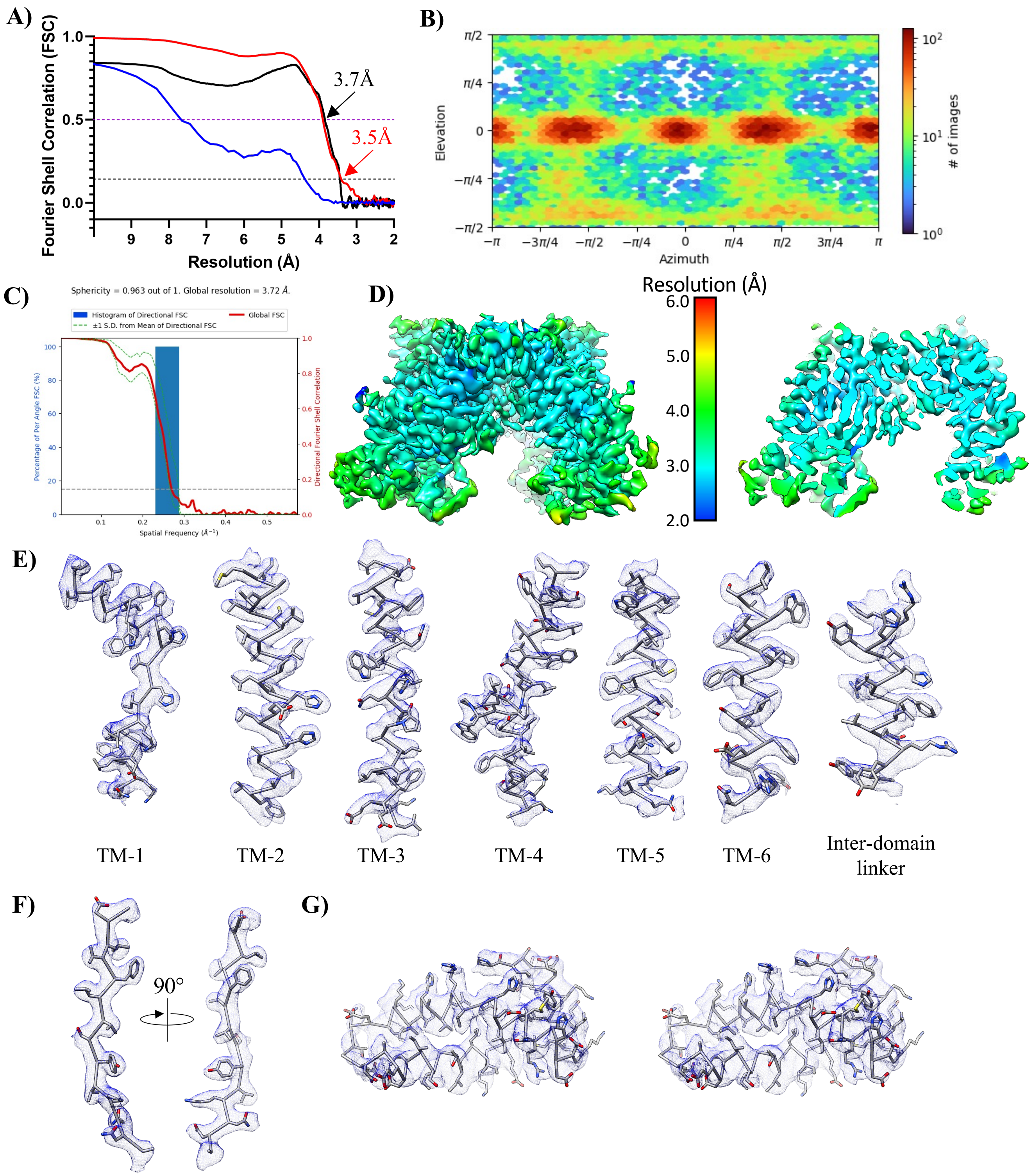

Supplemental Figure S3. CryoEM Data Analysis for WT SpoIVFB:Pro-σ^K Complex

A) FSC curve for the final 3D reconstruction of tetrameric WT SpoIVFB:Pro-σ^K. Blue curve shows the FSC between unmasked half-maps, red curve shows FSC between the same half-maps after applying a tight mask in CryoSPARC. The black curve represents FSC between the cryo-EM map and the final atomic model. Gold standard FSC=0.143 is indicated with a dotted black line, and FSC=0.5 is indicated with a dotted purple line. **B)** Angular distribution of particles in the final reconstruction of WT SpoIVFB:Pro-σ^K. **C)** 3D FSC as calculated from the 3DFSC web server. **D)** Local resolution map showing variation of resolution across the final 3D reconstruction. Two views show the entire map (left) and a clipped view to show internal resolution (right). **E)** Coulomb potential map for the TM helices in one SpoIVFB. **F)** Coulomb potential map for the pro-sequence of one Pro-σ^K subunit. **G)** Stereo-view of the coulomb potential map in the soluble domain of Pro-σ^K.

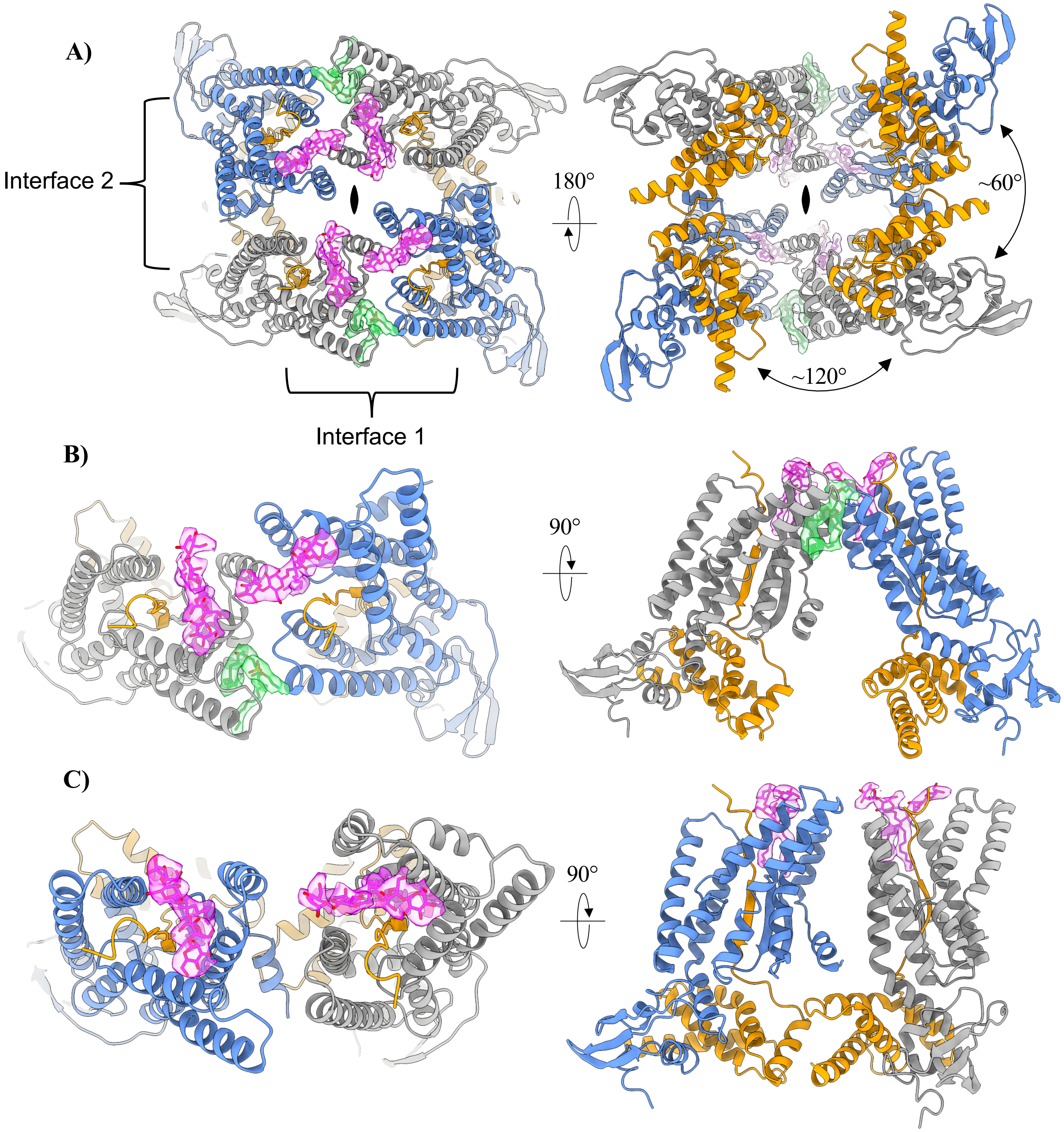

Supplemental. Figure S4. Interfaces in Tetrameric SpoIVFB:Pro-σ^K

A) Cartoon representation of the tetrameric WT SpoIVFB:Pro-σ^K assembly. SpoIVFB is colored blue or grey, Pro-σ^K is colored orange, LMNG detergent is colored magenta, and a lipid-like density is colored green. The twofold axis of symmetry is indicated with a black symbol. Two different interfaces between SpoIVFB:Pro-σ^K monomers are indicated with brackets. **B)** Views of interface 1 between monomers as indicated in **A**. Interface 1 is largely formed by interactions between SpoIVFB from individual monomers and LMNG detergent molecules and a lipid. **C)** Views of interface 2 between monomers as indicated in **A**. Interface 2 is largely formed by interactions between Pro-σ^K from individual monomers, with limited physical interaction between SpoIVFB from individual monomers.

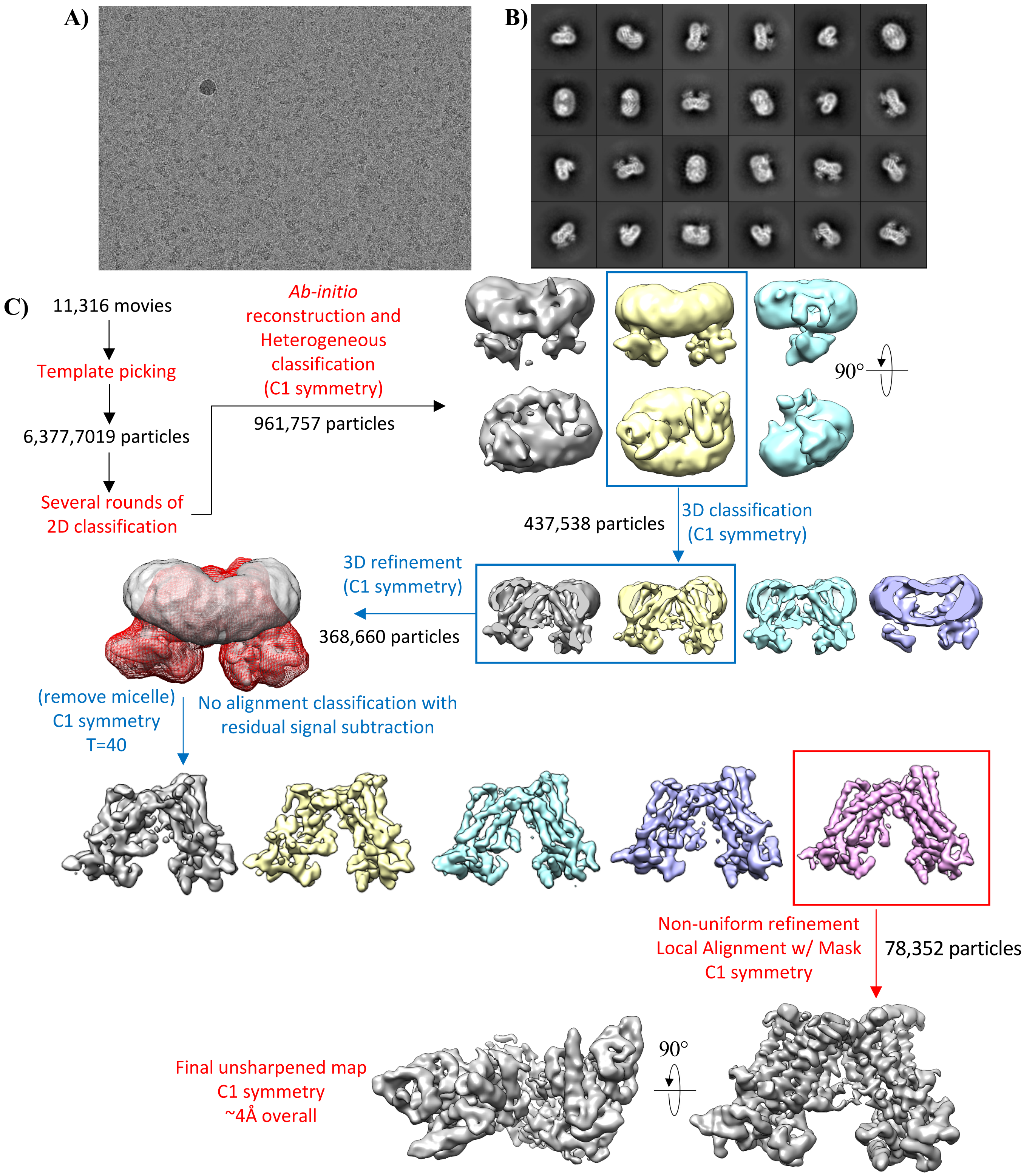

Supplemental Figure S5. Cryo-EM Processing for E44Q SpoIVFB:Pro-σ^{K} Complex
A) Representative electron micrograph from a dataset of 11,316 micrographs showing the distribution of E44Q SpoIVFB:Pro-σ^{K} particles in ice. **B)** 2D class-averages of the E44Q SpoIVFB:Pro-σ^{K} complexes. **C)** Data processing scheme to reconstruct the dimeric assembly of E44Q SpoIVFB:Pro-σ^{K} complexes. Steps shown in red were performed in CryoSPARC and steps shown in blue were performed in Relion. Particles numbers in each step are written in black.

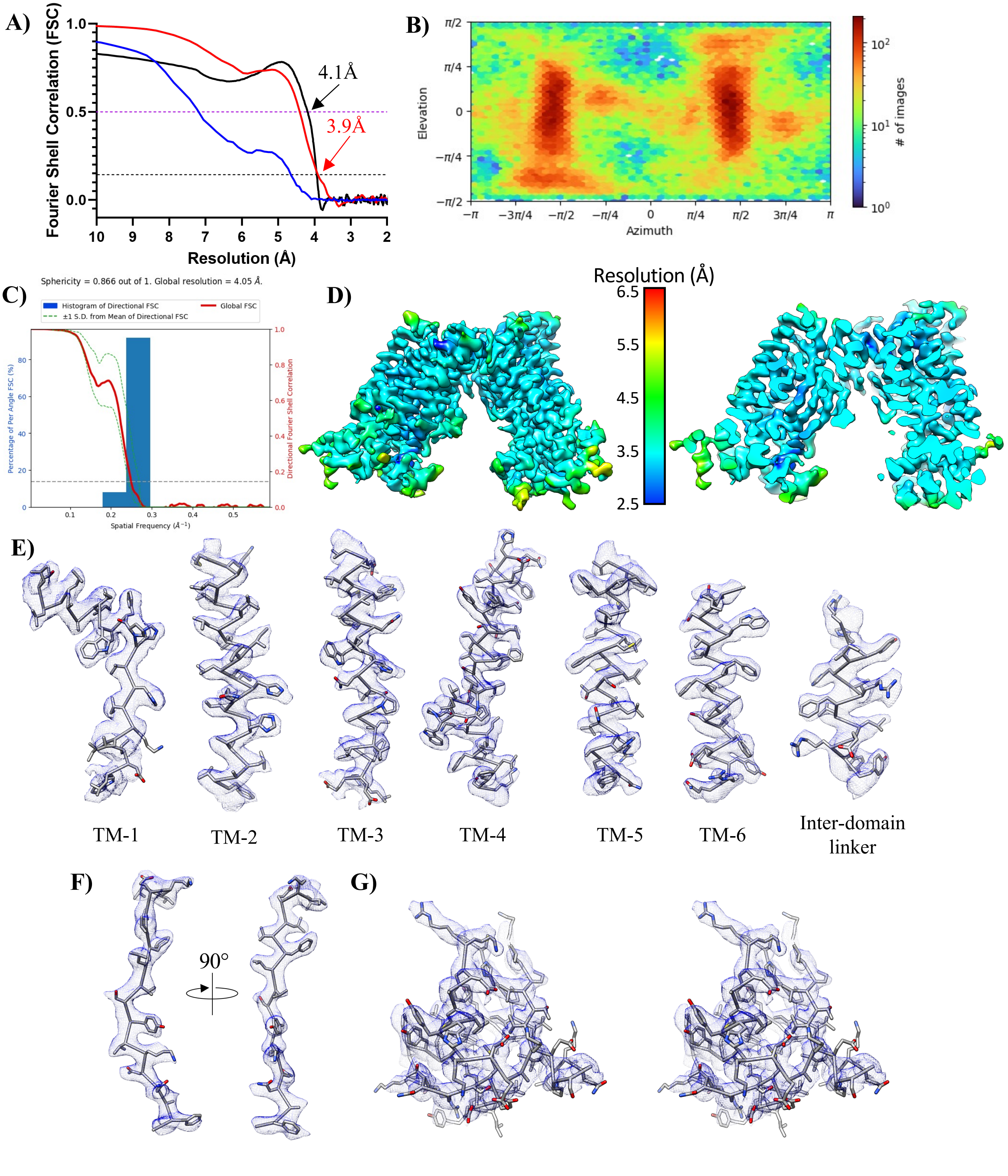

Supplemental Figure S6. Cryo-EM Data Analysis for E44Q SpoIVFB:Pro-σ^K Complex

A) FSC curve for the final 3D reconstruction of dimeric E44Q SpoIVFB:Pro-σ^K. Blue curve shows the FSC between unmasked half-maps, red curve shows FSC between the same half-maps after applying a tight mask in CryoSPARC. The black curve represents FSC between the cryo-EM map and the final atomic model. Gold standard FSC=0.143 is indicated with a dotted black line, and FSC=0.5 is indicated with a dotted purple line. **B)** Angular distribution of particles in the final reconstruction of E44Q SpoIVFB:Pro-σ^K. **C)** 3D FSC as calculated from the 3DFSC web server. **D)** Local resolution map showing variation of resolution across the final 3D reconstruction. **E)** Coulomb potential map for the TM helices in one SpoIVFB. **F)** Coulomb potential map for the pro-sequence of one Pro-σ^K subunit. **G)** Stereo-view of the coulomb potential map in the CBS domain of one SpoIVFB subunit.

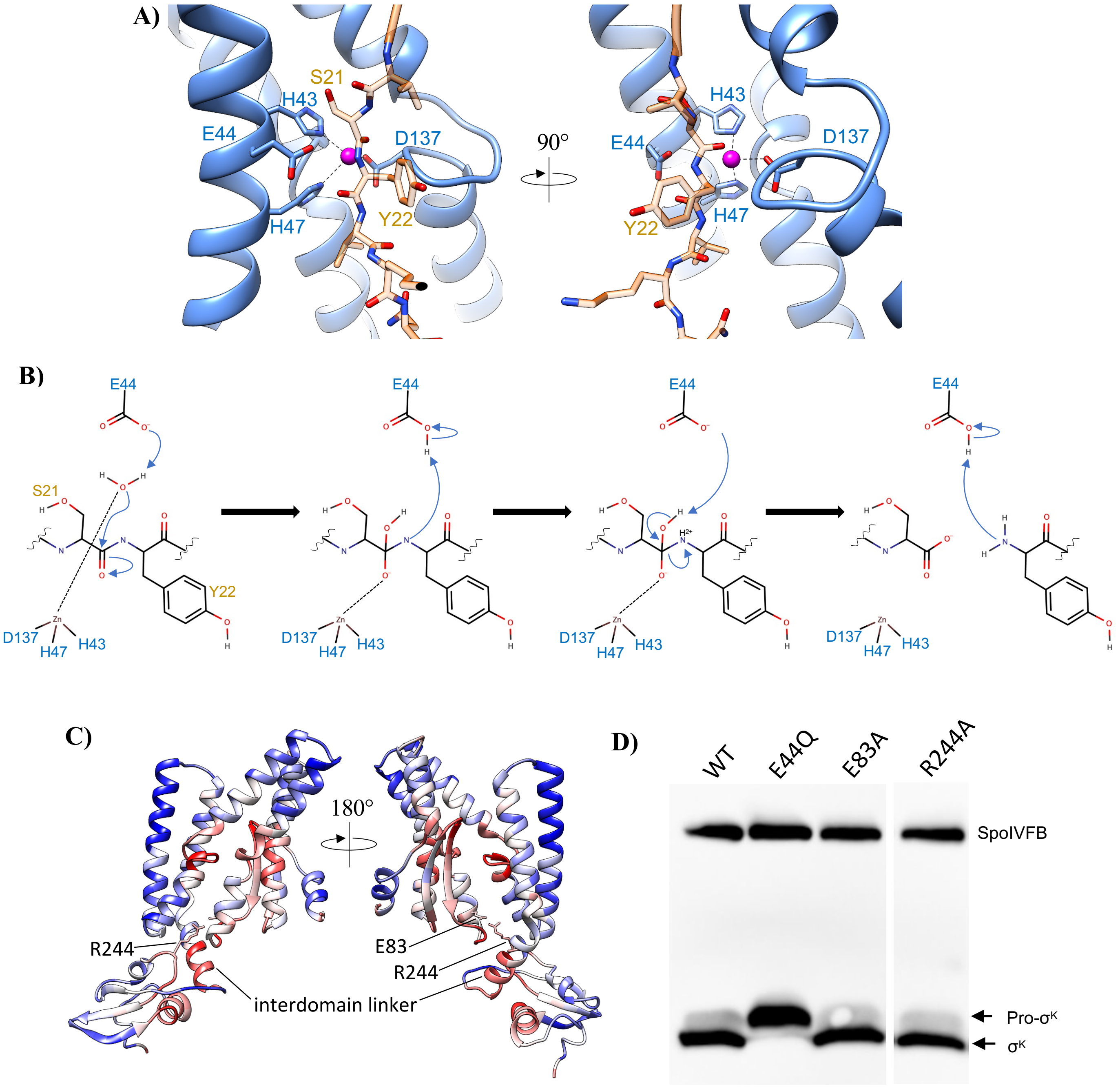

Supplemental Figure S7. Comparison of Structures and Proposed Zinc-Dependent Proteolysis Mechanism

A) View of the SpoIVFB active site as observed in the cryo-EM structure of WT SpoIVFB:Pro-σ^K. The zinc ion (magenta sphere) was not observed in the cryo-EM structure but is modeled here for clarity. The carbonyl in the peptide backbone of Pro-σ^K between residue S21 and Y22 points towards the zinc-binding pocket based on the position of the zinc ion in the mjS2P structure. **B)** Proposed catalytic mechanism of SpoIVFB-mediated intramembrane proteolysis of Pro-σ^K. SpoIVFB residues H43, H47, and D137 coordinate a Zn^{2+} ion and E44 acts as a base to activate a water molecule for nucleophilic attack on the carbonyl of the peptide bond located between Pro-σ^K residues S21 and Y22. The bound zinc ion stabilizes the oxyanion intermediate that develops on the peptide backbone carbonyl following nucleophilic attack. **C)** Plot of sequence conservation across the structure of SpoIVFB. Red indicates regions of high sequence conservation, and blue indicates regions of low sequence conservation. **D)** Cleavage assay of SpoIVFB mutants showing that mutation of the conserved residue E83 or R244 to alanine has no effect on Pro-$\boldsymbol{\sigma}^K$ processing.

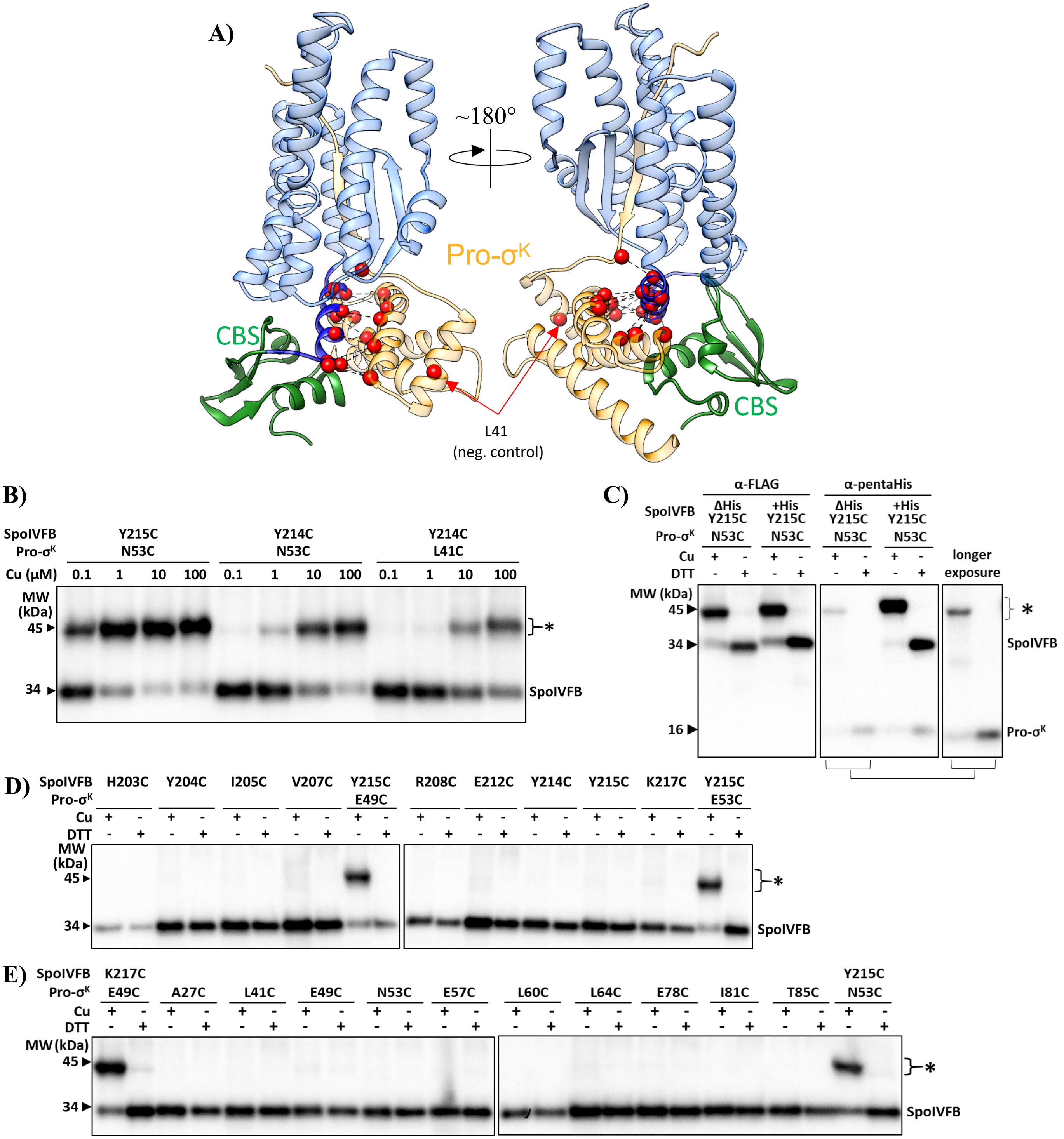

Supplemental Figure S8. Disulfide Crosslinking Analysis of SpoIVFB:Pro-σ^K

A) Two rotated views of SpoIVFB:Pro-σ^K with positions of cysteine variants for disulfide crosslinking highlighted with red spheres. Pairs of cysteine residues to test crosslinking are shown with dotted black lines. Pro-σ^K residue L41 that was used as a negative control is highlighted. **B)** Anti-FLAG western blot showing titration of oxidant Cu^{2+}(phenanthroline)$_3$ (Cu) concentration for three different pairs of SpoIVFB and Pro-σ^K single-cysteine variants subjected to *in vivo* disulfide crosslinking. The position of cysteine residues in SpoIVFB and Pro-σ^K is indicated at the top, as is the Cu concentration. Crosslinked SpoIVFB species are indicated with an asterisk. The Y214C-L41C pair serves as a negative control with a predicted distance of ~20 Å in the cryo-EM structure, and only crosslinks at higher Cu concentrations. **C)** Western blots showing both SpoIVFB and Pro-σ^K are in the crosslinked species. SpoIVFB Y215C with (+His) or without (ΔHis) a His$_6$-tag was subjected to *in vivo* disulfide crosslinking with His$_6$-tagged Pro-σ^K N53C. A blot with duplicate samples was cut in two and probed with anti-FLAG or anti-pentaHis antibodies. The Pro-σ^K signal was weak compared to that of SpoIVFB on the anti-pentaHis blot, so a longer exposure of part of the blot is shown to highlight that the majority of Pro-σ^K is in the crosslinked species indicated with an asterisk. **D)** Anti-FLAG western blots showing cysteine-less Pro-σ^K (blank lanes) does not disulfide crosslink with single-cysteine SpoIVFB variants. **E)** Anti-FLAG western blots showing cysteine-less SpoIVFB (blank lanes) does not disulfide crosslink with single-cysteine Pro-σ^K variants. The position of migration of protein molecular weight (MW) markers is shown in **B-E**.

Supplemental Figure S9. MD analysis of the WT SpoIVFB:Pro-σ^K Complex

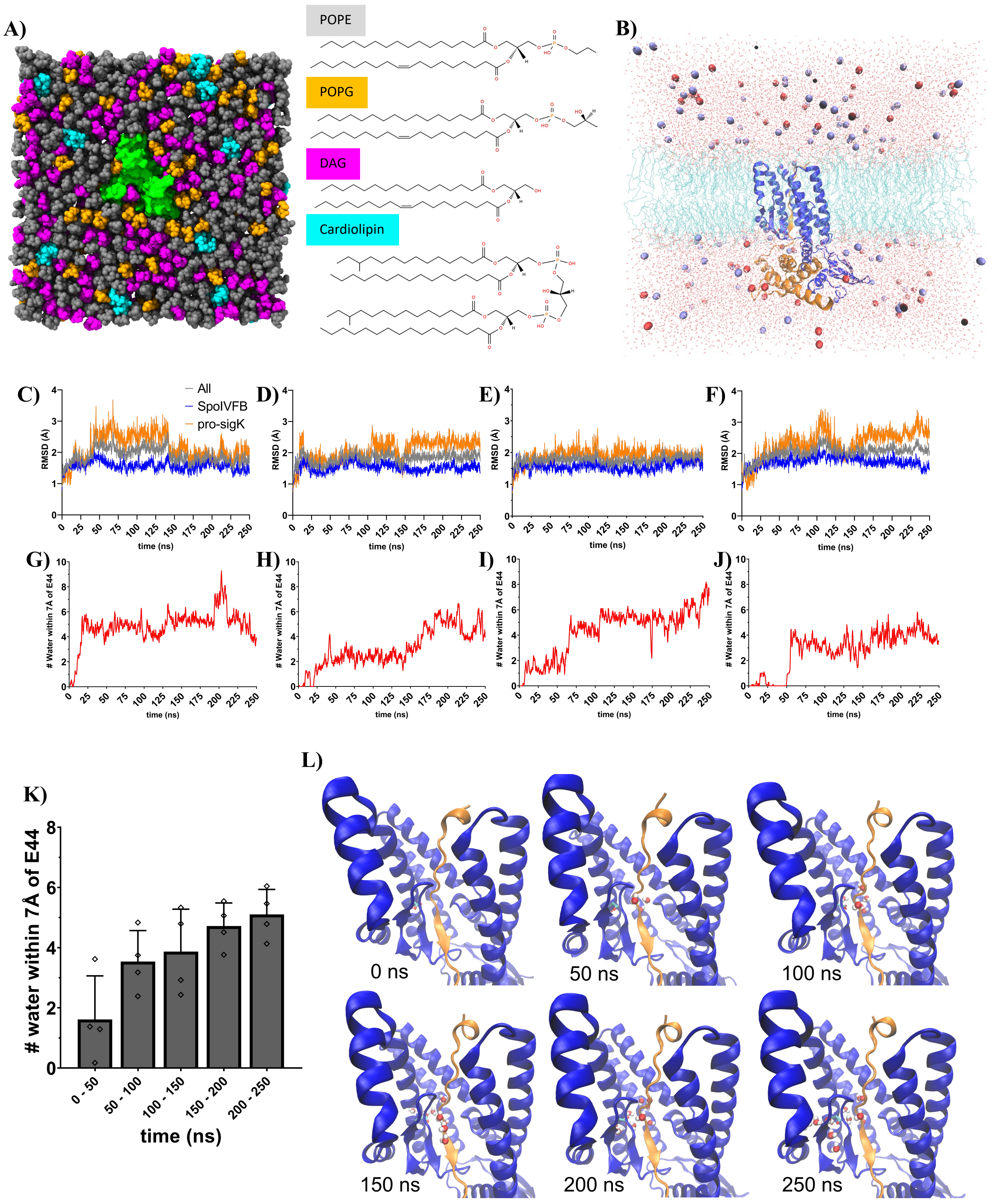

Supplemental Figure S9. MD analysis of the WT SpoIVFB:Pro-σ^K Complex

A) Membrane lipid composition of the simulations. Individual lipids are color coded and their structures are shown to the right. The SpoIVFB:Pro-$\boldsymbol{\sigma}^K$ complex embedded in the bilayer is shown as a green surface as viewed from the intermembrane space. **B)** View of a simulation box after equilibration and before unrestrained MD simulation. SpoIVFB is colored blue, Pro-σ^K is colored orange, lipids are colored cyan, waters are shown as red dots, and potassium and chloride ions are shown as spheres. **C-F)** RMSD plots of SpoIVFB and Pro-σ^K over the course of four replicate 250 ns simulations. SpoIVFB is shown as a blue trace, Pro-σ^K is shown as an orange trace, and the average over all protein is shown as a grey trace. **G-J)** Moving average (over 10 frames) of water molecules within a 7Å distance of the SpoIVFB E44 side chain in four replicate simulations. **K)** Average number of water molecules within 7Å of the SpoIVFB E44 sidechain calculated across moving windows of time. Bars represent the average number of water molecules within the specified distance in each of the four (n=4) replicate simulations. Error bars represent SD across four measurements from replicate simulations. Open circles represent the measurement from each independent simulation. **L)** Visualization of water entry near the SpoIVFB active site in replicate simulation #3. SpoIVFB is colored blue, Pro-σ^K is colored orange, and water molecules are shown as red and white spheres.

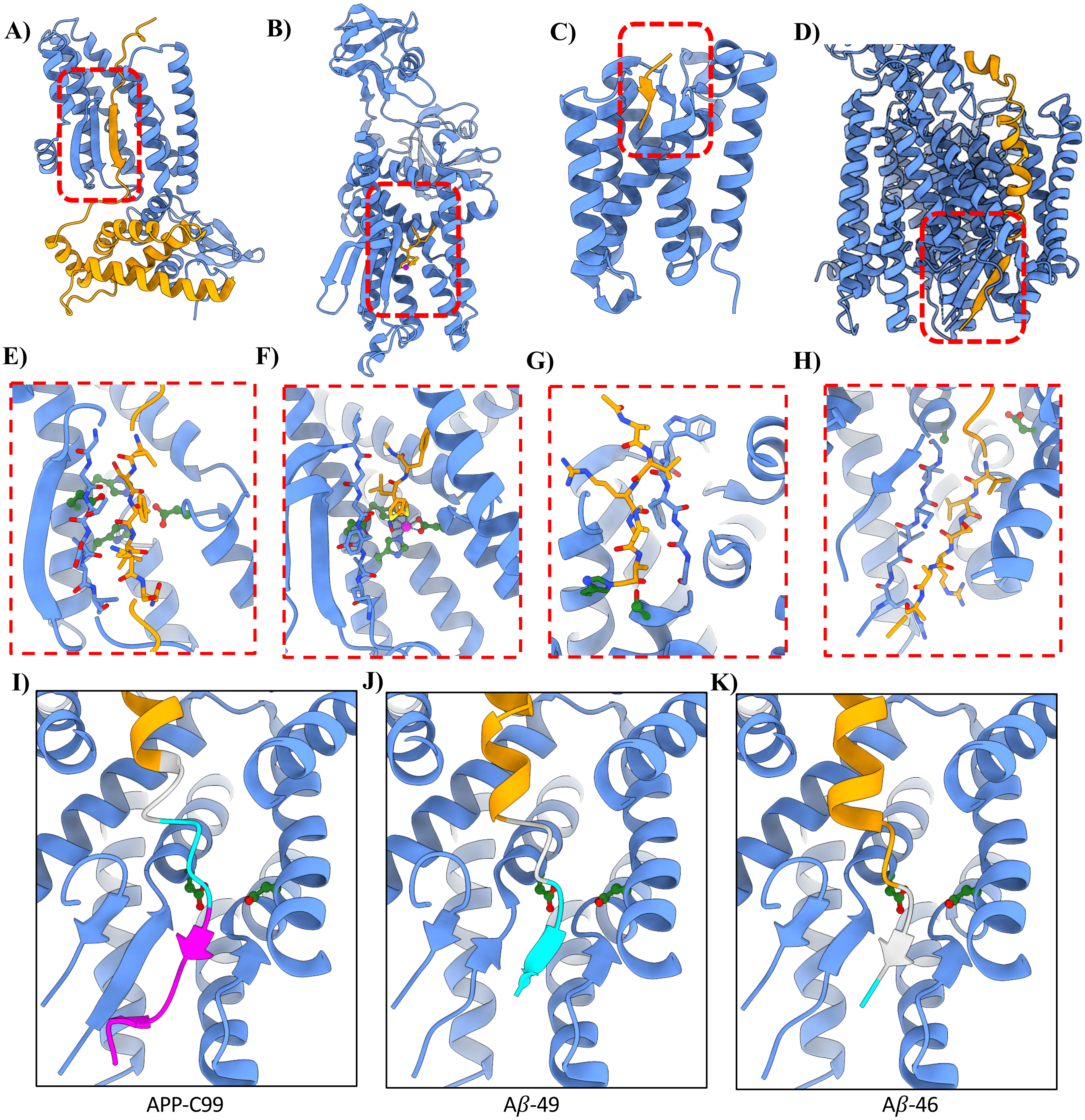

Supplemental Figure S10. *β*-sheet augmentation is a common mechanism of substrate/ligand binding in intramembrane proteases

A-D) Shown are cartoon representations of (**A**) SpoIVFB bound to the substrate Pro-σ^K (PDB = 8VJL), (**B**) RseP bound to the peptide mimetic inhibitor batimistat (PDB = 7W6X) (**C**) GlpG bound to a peptide mimetic inhibitor (PDB = 5MT8), (**D**) gamma-secretase bound to the substrate Notch (PDB = 6IDF). Enzymes are colored blue and substrates/inhibitors are colored orange. The enzyme active site with bound substrate/inhibitor engaging through beta-sheet augmentation is highlighted in a dashed red box. **E-H)** Zoomed in views of the regions highlighted above in **A-D**. The interacting beta-strands of substrate/inhibitor and enzyme are shown as sticks, revealing a beta-sheet architecture of alternating backbone hydrogen bonds. Active site residues are shown in green spheres. **I-J*)*** View of the human gamma-secretase complex active site with **I)** APP-C99 (PDB = 8X54), **J)** A*β*-49 (PDB = 8X52), or **K)** A*β*-46 (PDB = 8X53) bound. Presenilin-1 is shown as a blue cartoon, with active site aspartic acid residues shown as green ball and stick. Substrates are colored according to successive cleavage sites (magenta, cyan, grey, orange)

Supplemental Table S1. Cryo-EM data collection, refinement, and validation statistics

	WT SpoIVFB:pro-σ^K (EMDB-43288) (PDB 8VJL)	E44Q SpoIVFB:pro-σ^K (EMDB-43289) (PDB 8VJM)
**Data collection and processing**		
Magnification	105,000	105,000
Voltage (kV)	300	300
Electron exposure (e–/Å^2)	60.5	50
Defocus range (μm)	0.7-2.5	0.8-2.0
Pixel size (Å)	0.872	0.849
Symmetry imposed	C2	C1
Initial particle images (no.)	6,595,786	6,377,7019
Final particle images (no.)	36,371	78,352
Map resolution (Å)	3.5	4.0
FSC threshold	0.143	0.143
**Refinement**		
Model resolution (Å)	3.7	4.1
FSC threshold	0.5	0.5
Map sharpening *B* factor (Å^2)	96.4	149.0
Model composition		
Non-hydrogen atoms	13338	6669
Protein residues	1616	808
Ligands	6	3
B factors (Å^2)		
Protein	50.6	77.6
Ligand	28.9	55.6
R.m.s. deviations		
Bond lengths (Å)	0.003	0.003
Bond angles (°)	0.563	0.634
Validation		
MolProbity score	1.80	1.88
Clashscore	6.97	8.59
Poor rotamers (%)	0.00	0.29
Ramachandran plot		
Favored (%)	93.69	93.75
Allowed (%)	5.81	6.12
Disallowed (%)	0.50	0.12
