# Supplementary material for: Substrate engagement by the intramembrane metalloprotease SpoIVFB
Source: Nat Commun. 2024 Oct 17;15:8276. doi: 10.1038/s41467-024-52634-6 (PMC11486902; doi:10.1038/s41467-024-52634-6)
Supplement: Supplementary file 3 — Description of Additional Supplementary Files [file 41467_2024_52634_MOESM3_ESM.pdf]

Description of Additional Supplementary Files

File Name: Supplementary Movie 1

Description: Penetrance of water near the SpoIVFB active site

Shown is the first 50 ns of simulation time after releasing equilibration restraints for simulation replica 3. SpoIVFB is colored transparent blue, Pro-sK is colored orange, zinc coordinating active site residues are shown as green sticks, and SpoIVFB residues E83 and R244 forming an electrostatic lock at the bottom of the TM helices is shown as magenta sticks. Shortly after releasing equilibration restraints water begins to penetrate from the mother cell cytoplasm (bottom) up through the region of the electrostatic lock highlighted in magenta.
